# Regional Contingencies in the Relationship between Aboveground Biomass and Litter in the World’s Grasslands

**DOI:** 10.1371/journal.pone.0054988

**Published:** 2013-02-06

**Authors:** Lydia R. O’Halloran, Elizabeth T. Borer, Eric W. Seabloom, Andrew S. MacDougall, Elsa E. Cleland, Rebecca L. McCulley, Sarah Hobbie, W. Stan Harpole, Nicole M. DeCrappeo, Chengjin Chu, Jonathan D. Bakker, Kendi F. Davies, Guozhen Du, Jennifer Firn, Nicole Hagenah, Kirsten S. Hofmockel, Johannes M. H. Knops, Wei Li, Brett A. Melbourne, John W. Morgan, John L. Orrock, Suzanne M. Prober, Carly J. Stevens

**Affiliations:** 1 Department of Zoology, Oregon State University, Corvallis, Oregon, United States of America; 2 Department of Ecology, Evolution, and Behavior, University of Minnesota, St. Paul, Minnesota, United States of America; 3 Department of Integrative Biology, University of Guelph, Guelph, Ontario, Canada; 4 Ecology, Behavior and Evolution Section, University of California San Diego, La Jolla, California, United States of America; 5 Department of Plant and Soil Sciences, University of Kentucky, Lexington, Kentucky, United States of America; 6 Department of Ecology, Evolution and Organismal Biology, Iowa State University, Ames, Iowa, United States of America; 7 U.S. Geological Survey, Forest and Rangeland Ecosystem Science Center, Corvallis, Oregon, United States of America; 8 MOE Key Laboratory of Arid and Grassland Ecology, School of Life Sciences, Lanzhou University, Lanzhou, China; 9 School of Environmental and Forest Sciences, University of Washington, Seattle, Washington, United States of America; 10 Department of Ecology and Evolutionary Biology, University of Colorado, Boulder, Colorado, United States of America; 11 Queensland University of Technology, Faculty of Science and Engineering, School of Earth, Environment and Biological Sciences, Brisbane, Queensland, Australia; 12 School of Life Sciences, University of KwaZulu-Natal, Scottsville, South Africa; 13 School of Biological Sciences, University of Nebraska, Lincoln, Nebraska, United States of America; 14 Department of Botany, La Trobe University, Bundoora, Victoria, Australia; 15 Zoology Department, University of Wisconsin, Madison, Wisconsin, United States of America; 16 CSIRO Ecosystem Sciences, Wembley, Western Australia, Australia; 17 Department of Environment, Earth and Ecosystems, The Open University, Milton Keynes, United Kingdom; 18 Department of Ecology and Evolutionary Biology, Yale University, New Haven, Connecticut, United States of America; 19 Lancaster Environment Centre, Lancaster University, Lancaster, United Kingdom; Lakehead University, Canada

## Abstract

Based on regional-scale studies, aboveground production and litter decomposition are thought to positively covary, because they are driven by shared biotic and climatic factors. Until now we have been unable to test whether production and decomposition are generally coupled across climatically dissimilar regions, because we lacked replicated data collected within a single vegetation type across multiple regions, obfuscating the drivers and generality of the association between production and decomposition. Furthermore, our understanding of the relationships between production and decomposition rests heavily on separate meta-analyses of each response, because no studies have simultaneously measured production and the accumulation or decomposition of litter using consistent methods at globally relevant scales. Here, we use a multi-country grassland dataset collected using a standardized protocol to show that live plant biomass (an estimate of aboveground net primary production) and litter disappearance (represented by mass loss of aboveground litter) do not strongly covary. Live biomass and litter disappearance varied at different spatial scales. There was substantial variation in live biomass among continents, sites and plots whereas among continent differences accounted for most of the variation in litter disappearance rates. Although there were strong associations among aboveground biomass, litter disappearance and climatic factors in some regions (e.g. U.S. Great Plains), these relationships were inconsistent within and among the regions represented by this study. These results highlight the importance of replication among regions and continents when characterizing the correlations between ecosystem processes and interpreting their global-scale implications for carbon flux. We must exercise caution in parameterizing litter decomposition and aboveground production in future regional and global carbon models as their relationship is complex.

## Introduction

It is a long-held tenet of ecosystem ecology that regional (*i.e.*, areas bounded by sub-continental scale geographic features) variation in production and decomposition processes are positively correlated with both temperature and precipitation and hence, production and decomposition processes should be coupled at regional scales, e.g. [1]–[3]. This assumption is supported by recent meta-analyses and models that suggest climate strongly influences plant production and decomposition rates of terrestrial foliage [4]–[7]. Carbon cycling models (*e.g.,* CENTURY model [8], [9]), motivated by such results, assume a coupling between net primary production (NPP) and litter loss, driven by parallel responses to temperature and precipitation. Given predicted scenarios of climate change, these carbon models predict significant changes to the way that biological systems influence atmospheric carbon dioxide concentrations [10], [11]. The degree of coupling will be particularly important for regions where live biomass and litter accumulation are not in equilibrium.

A challenge to understanding and quantifying the production-decomposition relationship is considering the covarying influence of other regulatory factors. Biotic drivers such as vegetation type, vegetation chemistry, and trophic interactions can also significantly affect rates of plant growth or organic matter decay, even within the same climatic region (*e.g.,* [4], [12], [13], [6], [14], [15]. Because production and decomposition are rarely measured concurrently, and because these processes are often characterized across large spatial scales where vegetative type covaries with climate, the relative effects of biotic and climate drivers can be difficult to untangle [16], [17]. Further, abiotic drivers other than temperature and precipitation also influence plant growth and litter decomposition, including nutrient limitation [18]–[20] and UV degradation in semi-arid environments [21]. The net result is that climate impacts on production and decomposition, rather than being universal, could vary regionally depending on the relative strength of these other factors. Testing for regional variation in the relationship between production and decomposition is crucial to climate change research globally because it may require revisions to ecosystem response projections that inform Earth system models.

Here we test whether climate factors (precipitation, temperature, radiation), elevation, and latitude predict concurrent aboveground biomass (as an estimate of aboveground net primary production) and litter disappearance (as an estimate of litter decomposition) in grassland ecosystems worldwide. Recent global syntheses have shown that plant functional traits play a major role in influencing decomposition rates [6], so we examine drivers of aboveground biomass and litter disappearance within ecosystems dominated by herbaceous species (mainly members of the Poaceae family) to control for functional composition. We also focus on this biome because grasslands are globally important in terms of carbon pools, species diversity, and human livelihood. Grasslands cover approximately 30% of the Earth’s ice-free surface and are critical for supporting livestock and maintaining biodiversity [22]. Further, the relative rates of production and decomposition in this biome control soil carbon pools, and govern whether these systems are a carbon source or sink [23]–[26]. Thus, accurately parameterized models of grassland production and decomposition using such data will be useful in predicting potential feedbacks in grasslands under future climate scenarios.

Grassland aboveground biomass and litter loss may vary at small spatial scales (<1 km) due to species interactions such as plant species competition for resources, interactions with the microbial community, herbivore density, or soil and plant chemistry. These processes also may vary at larger regional or continental scales due to climatic and/or environmental factors. We hypothesized that aboveground biomass and litter disappearance are positively correlated at smaller plot and site scales because of similarities in species pools and abiotic conditions. Factors that could limit the amount of biomass production, *e.g.,* low temperatures, radiation and precipitation, will also limit amount of loss through decomposition, thus making them positively correlated. Likewise, sites that have high biomass production should have high rates of loss. We also hypothesized that the greatest amount of variation in aboveground biomass and loss should be found at the regional or continental scale due to differences in climate.

## Methods

### Site Selection

Our study included data from 39 sites that are part of the globally-distributed Nutrient Network (http://nutnet.org/). Access to study areas was negotiated by the lead scientist at each site. All sites are dominated by low-statured, primarily grassland vegetation. Each site selected for the study is relatively homogeneous (*i.e.*, not encompassing large or obvious environmental gradients) and dominated by herbaceous vegetation, primarily Poaceae. Sites actively grazed by livestock or burned for management purposes were excluded from this study. Most sites sampled vegetation in 2007, but a subset sampled in 2008. The sites in this study range from 37.81°S to 53.99°N latitude, 250 to 2314 mm year^−1^ mean annual precipitation, 0 to 22.1°C mean annual temperature and 0.5 to 3500 m in elevation. Sites were located in Australia, Canada, China, Germany, South Africa, Switzerland, Tanzania and the United States (Table 1). We included some anthropogenic grassland sites (i.e. historically altered by humans via fire or clearing to create grass dominance), given the increasing prevalence of these grasslands globally [27]. There were no statistical differences between natural and anthropogenic grasslands for any of our measures (results not shown), so we include all sites as one dataset.

**Table 1:** Nutrient Network experimental sites.

Site	Country	State	Region	Latitude	Longitude	Elevation (m)	MAP (mm)	MAT (C)
American Camp	USA	Washington	Pacific Coast	48.47	–123.01	41	672.4	9.8
Azi	China	Gansu	Eurasia	33.58	101.53	3500	620.0	0
Barta Brothers	USA	Nebraska	Great Plains	42.24	−99.65	767	568.0	8.7
Bogong	Australia	Victoria	Australia	−36.87	147.25	1760	1217.0	5.7
Boulder	USA	Colorado	Great Plains	39.97	−105.23	1633	482.0	9.7
Bunchgrass LTER	USA	Oregon	IM West	44.28	−122.26	1318	2160.0	5.5
Burrawan	Australia	Queensland	Australia	27.73	151.14	425	600.0	18.4
Buttercup LTER	USA	Oregon	IM West	44.28	−121.96	1500	2160.0	5
Cedar Creek LTER	USA	Minnesota	Great Plains	45.40	−93.20	270	800.0	6.3
Cedar Point	USA	Nebraska	Great Plains	41.20	−101.63	965	470.0	9.3
Chichaqua Bottoms	USA	Iowa	Great Plains	41.79	−93.39	275	891.0	9
Cowichan	Canada	British Columbia	Pacific Coast	48.46	123.38	50	1038.6	9.8
Finley	USA	Oregon	Pacific Coast	44.41	−123.28	68	1200.0	11.3
Glacial Heritage	USA	Washington	Pacific Coast	46.87	−123.03	33	1299.8	10.5
Hall’s Prairie	USA	Kentucky	Great Plains	36.96	−86.73	194	1282.0	13.6
Hanover	USA	New Hampshire	Atlantic Coast	43.42	−72.14	271	919.5	6.4
Hart Mountain	USA	Oregon	IM West	42.72	−119.50	1508	304.8	7.4
Hastings	USA	California	Pacific Coast	36.20	−121.55	750	550.0	10.9
Hopland	USA	California	Pacific Coast	39.00	−123.07	417	939.8	12.3
Jasper Ridge	USA	California	Pacific Coast	37.41	−122.24	120	655.0	13.8
Kinypanial	Australia	Victoria	Australia	−36.20	143.75	90	395.0	15.5
Konza Prairie	USA	Kansas	Great Plains	39.08	−96.58	440	835.0	12
Leadbetter	USA	Washington	Pacific Coast	46.61	−124.05	2	2044.2	9.9
Lookout LTER	USA	Oregon	IM West	44.21	−122.26	1500	2314.0	4.8
Mclaughlin UCNRS	USA	California	Pacific Coast	38.87	−122.40	550	650.0	13.5
Mount Caroline	Australia	W. Australia	Australia	−31.78	117.61	285	352.0	17.3
Niwot LTER	USA	Colorado	IM West	39.99	−105.38	3050	930.0	6.4
Papenburg	Germany	Lower Saxony	Europe	53.09	7.47	0.5	850.1	8.9
Sagehen Creek UCNRS	USA	California	IM West	39.43	−120.24	1920	850.0	5.7
Savannah	USA	South Carolina	Atlantic Coast	33.34	81.65	71	1000.0	17.3
Sedgewick UCNRS	USA	California	Pacific Coast	34.70	−120.02	550	380.0	15
Serengeti	Tanzania	NA	Africa	−2.25	34.51	1536	789.0	22.1
Short−Grass LTER	USA	Colorado	Great Plains	40.82	−104.77	1650	341.7	8.4
Sierra Foothills	USA	California	Pacific Coast	39.29	−121.34	333	711.2	15.6
Smith Prairie	USA	Washington	Pacific Coast	48.21	−122.62	62	549.9	9.8
Tyson	USA	Missouri	Great Plains	38.52	90.56	169	1090.0	12.5
Ukulinga	South Africa	KwaZulu-Natal	Africa	−29.67	30.4	843	838.0	18.1
UNC-Duke	USA	North Carolina	Atlantic Coast	35.91	−79.06	141	1210.0	14.7
Val Mustair	Switzerland	NA	Europe	46.63	10.37	2329	950.0	0.3

Note: IM West = Intermountain West. Complete site names can be found at: www.nutnet.umn.edu/field_sites.

### Aboveground Biomass and Litter

**Figure 1:**
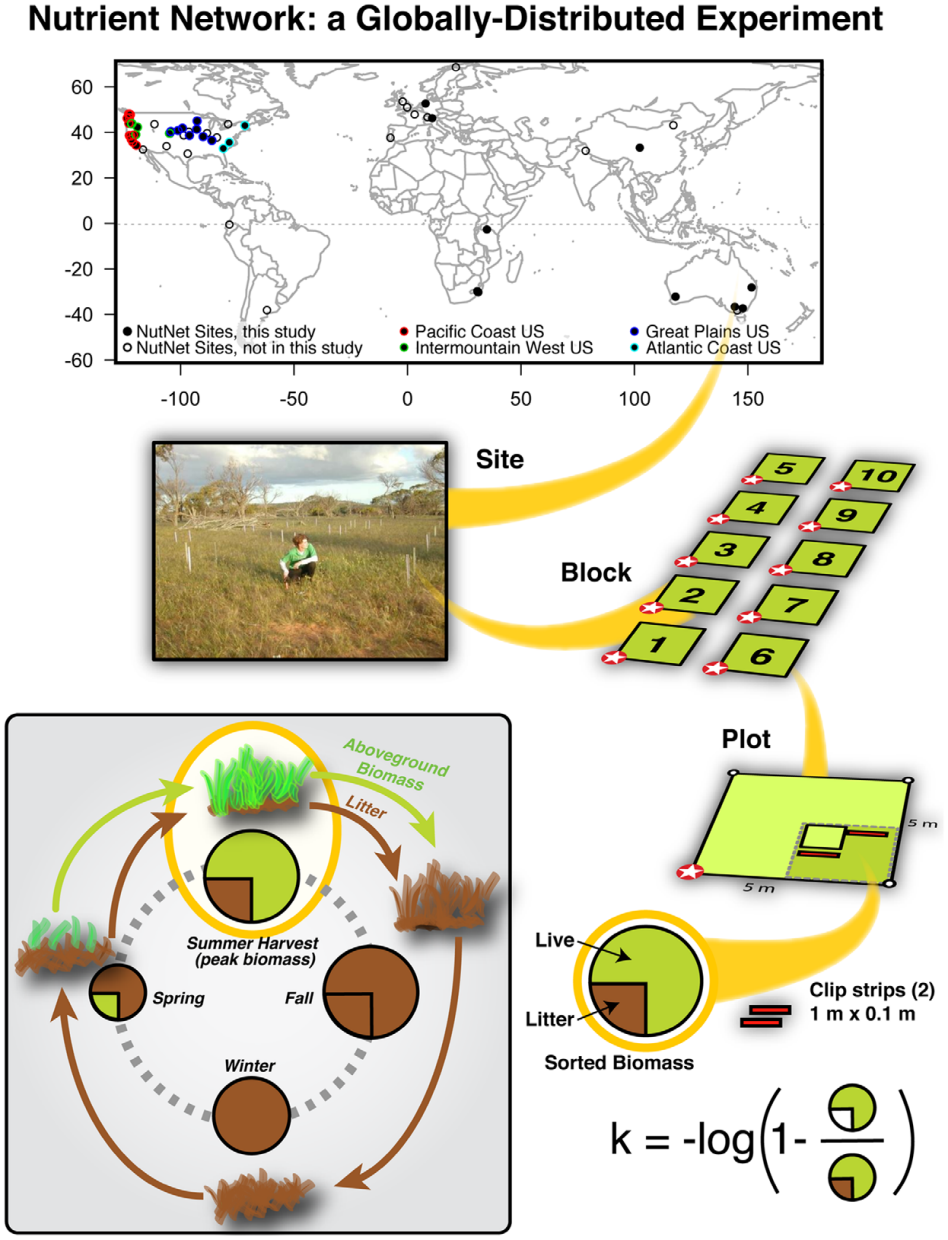
The Nutrient Network is a globally-distributed experiment testing top-down and bottom-up controls over grassland diversity and ecosystem function. Our nested hierarchical analysis quantified variability for aboveground biomass and litter disappearance for 39 sites among continents, regions (i.e., among sites in the continental US, shown as filled points with colored circles), sites, blocks within sites (each with 1–6 blocks of 8–10 plots per block), and plots within blocks. Aboveground biomass was sampled using identical protocols within a subplot of each plot and sorted to live (current year’s production) and litter (previous years’ production). Litter disappearance represents an estimate of the log-transformed fraction of the previous year’s total above ground biomass (live plus dead) that is remaining at the end of the subsequent growing season (litter biomass divided by total biomass) using Olson’s equation. The inset figure illustrates the fate of biomass over one growing season: Current year’s production (green) at end of growing season (Fall) senesces and combines with previous years’ production (brown); total litter biomass decays over time (indicated by decreasing size of circle); new production (green) in Spring increases while remaining litter continues to decrease; peak biomass along with remaining litter is harvested at the end of Summer and used to estimate litter disappearance rate (k = −log(litter/total) ).

The standard Nutrient Network sampling protocol was followed at all sites. Plots were 5×5 m. The majority (33 of 39) of sites sampled 3 blocks of 10 plots per block; although 1 site had 1 block, 1 had 2, 1 had 4, 2 had 5, and 1 had 6. There was a 1 m buffer between each plot. Aboveground live biomass and litter were collected in each plot from a randomly selected 0.2 m^2^ (10×200 cm) strip at peak biomass (Figure 1). For sites exhibiting biphasic seasonal growth patterns, biomass was collected and summed for both peak periods. Aboveground live biomass of individual plants rooted within the strip was clipped at ground level, and all litter standing stock also was collected. For plots with shrubs and subshrubs rooted within the strip, leaves and current year’s woody growth were collected. All biomass was dried to a constant mass at 60°C and weighed to the nearest 0.01 g. In these herbaceous ecosystems with minimal perennial aboveground organs, aboveground biomass provides an estimate of aboveground net primary production (ANPP), although the estimate may be slightly lower than the true value of ANPP because of tissue turnover during the growing season [28].

Litter disappearance is a metric used to estimate the amount of litter lost via decomposition and herbivory among growing seasons. This metric is a commonly used tool in estimating loss [29] in grassland studies [30], [31]. Because it derives from the sampling of aboveground biomass, it is a relatively easy measure allowing for high replication not possible with litter bags. It also captures the potential influence of UV-mediated decomposition on aboveground litter that is increasingly recognized as an important factor in grasslands but cannot be accurately measured by litter bags (bag material shields litter from direct radiation).

Litter disappearance estimates (*k*) were calculated using an equation derived from Olson [32] for deciduous forest decay rates:
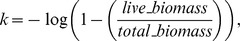
where *live biomass* is the standing stock during peak season and *total biomass* is live biomass plus litter collected at the same time (Figure 1). Although our experimental system is not a forested system as modeled in Olson’s paper, both are deciduous with annual biomass contributions to the litter pool.

### Temperature, Precipitation and Radiation Estimates

Precipitation and temperature data were generated from the WorldClim database [33]. We used four measures for each site (1 km^2^ scale resolution): mean annual temperature (MAT), mean annual precipitation (MAP), maximum summer temperature, and minimum winter temperature. The last two measures provide an estimate of temperature range at each site, given that both mean and variation in climate are known to affect growth and decomposition [34]. It is difficult to assess causation in observational data when there is strong covariance among the explanatory variables. In our case, climate variables were only weakly covarying with the exception of MAT and the derived minimum winter temperature where some degree of relationship would be expected. We derived a coefficient of variation from 10 years of precipitation data. Without commensurate biomass data, however, the analysis of interannual variability relationships was not possible.

Radiation data were generated from the NASA surface meteorology and solar energy release 6.0 data set (http://eosweb.larc.nasa.gov/sse/). A mean annual radiation was calculated for each site by integrating daily surface measurements (kWh/m^2^/day) over a 20-year period on a 1×1 degree grid.

### Statistical Methods

The relationship between aboveground biomass and litter disappearance was analyzed using a linear regression analysis both at the plot and site scale. We quantified variability for aboveground biomass and litter disappearance using variance component analyses in which continent, region, site, block, and plot were considered as nested random effects [35], [36]. We used a multiple linear regression to analyze the relationship between dependent (aboveground biomass, litter and litter disappearance) and independent variables (latitude, elevation, radiation, mean annual precipitation, mean annual temperature, mean minimum winter temperature and mean maximum summer temperature) at the site level. First order interactions between terms were also analyzed but no significance was found and interactions are not included in the results. A suite of non-linear relationships between independent and dependent variables were also explored using Eureqa [37] but no significant relationships were found and were not included in the results. In addition to the site-wide comparisons, the North American sites were divided into four regions based on the location of large mountain ranges (Pacific Coast, Intermountain West, Central, and Atlantic Coast). We also examined these relationships within three regions of the United States with sufficient replication for comparisons. All analyses were conducted using R version 2.8.0 [38].

## Results

**Figure 2:**
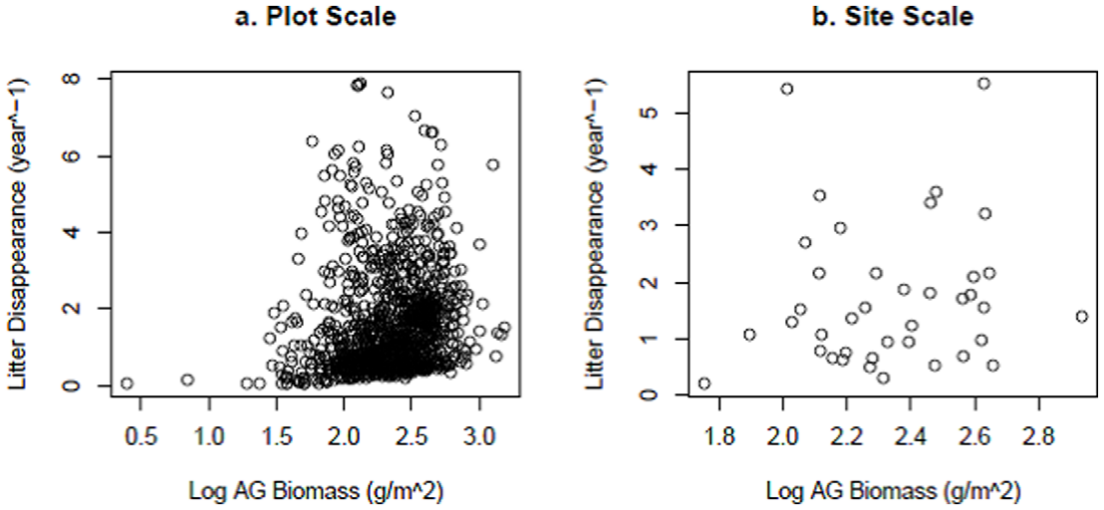
Aboveground (AG) biomass and litter disappearance were weakly correlated at the plot scale (a; *p*<0.0001, *r^2^* = 0.02) but not correlated at the site scale (b; *p* = 0.61, *r^2^* = 0.01).

Site scale biomass ranged from 61.5 g/m^2^ (Savannah River, Georgia, USA) to 917.8 g/m^2^ (Papenburg, Germany), and standing litter between sites ranged from 0.7 g/m^2^ (Mt. Caroline, Australia) to 689.6 g/m^2^ (Leadbetter, Washington, USA). Site scale litter disappearance ranged from 0.19 yr^−1^ (Savannah River, Georgia, USA) to 5.52 yr^−1^ (Ukulinga, South Africa), representing a larger range than for decomposition in North American grasslands (0.28 yr^−1^ to 1.73 yr^−1^ [39]). Aboveground biomass and litter disappearance showed a very weak positive relationship at the plot scale (*p*<0.0001, *r^2^* = 0.02; Figure 2a) but were not related when compared at the site scale (*p* = 0.61, *r^2^* = 0.01; Figure 2b).

**Figure 3:**
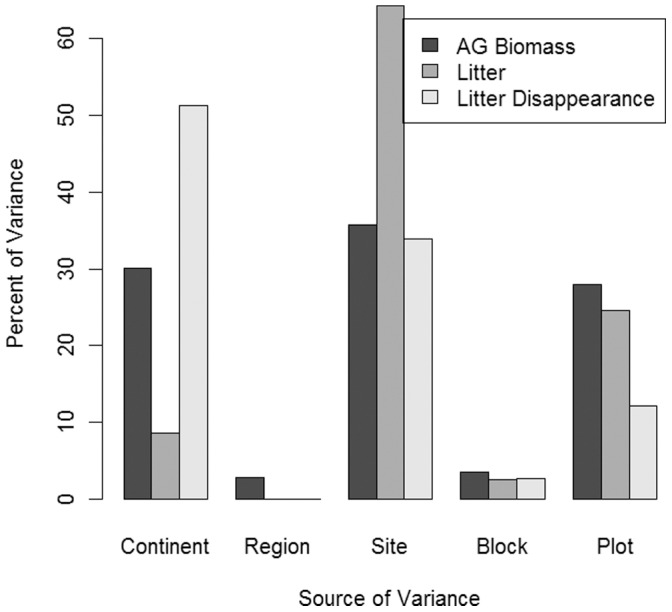
Variance components for site scale aboveground biomass, litter stocks, and litter disappearance.

Counter to our expectations, there were no strong correlations between site-level averages of aboveground biomass, litter, or litter disappearance and most climate variables (Table 2) at the site scale. Although there were some significant relationships (live biomass with radiation and latitude), the correlation coefficients were small, suggesting that climate variables are relatively poor predictors of aboveground biomass and loss across global scales. For example, radiation and latitude were correlated with biomass production across sites but were not correlated with litter or litter disappearance (Table 2). Litter disappearance and aboveground biomass also varied at different spatial scales (Figure 3); litter disappearance was strongly variable among continents, whereas variation in aboveground biomass was more evenly distributed across plots, sites and continents.

**Table 2:** Backwards selected multiple linear regression results for site-level live biomass model (R2 = 0.34, p<0.01).

Variable	Coefficient	Error	t	p
**Radiation**	−0.298	0.103	−2.89	0.01
**Latitude**	−0.022	0.010	−2.26	0.03
**Elevation**	–	–	–	–
**Max. High** 1	–	–	–	–
**Min. Low** 2	–	–	–	–
**MAT** 3	–	–	–	–
**MAP** 4	–	–	–	–

1 Maximum high temperature,

2 Minimum low temperature,

3 Mean annual temperature,

4 Mean annual precipitation.

– indicates non-significant terms and thus are not included in the final model or reported here. Note: Multiple linear regression analyses for litter and decomposition with climate variables were insignificant and not included in table.

**Figure 4:**
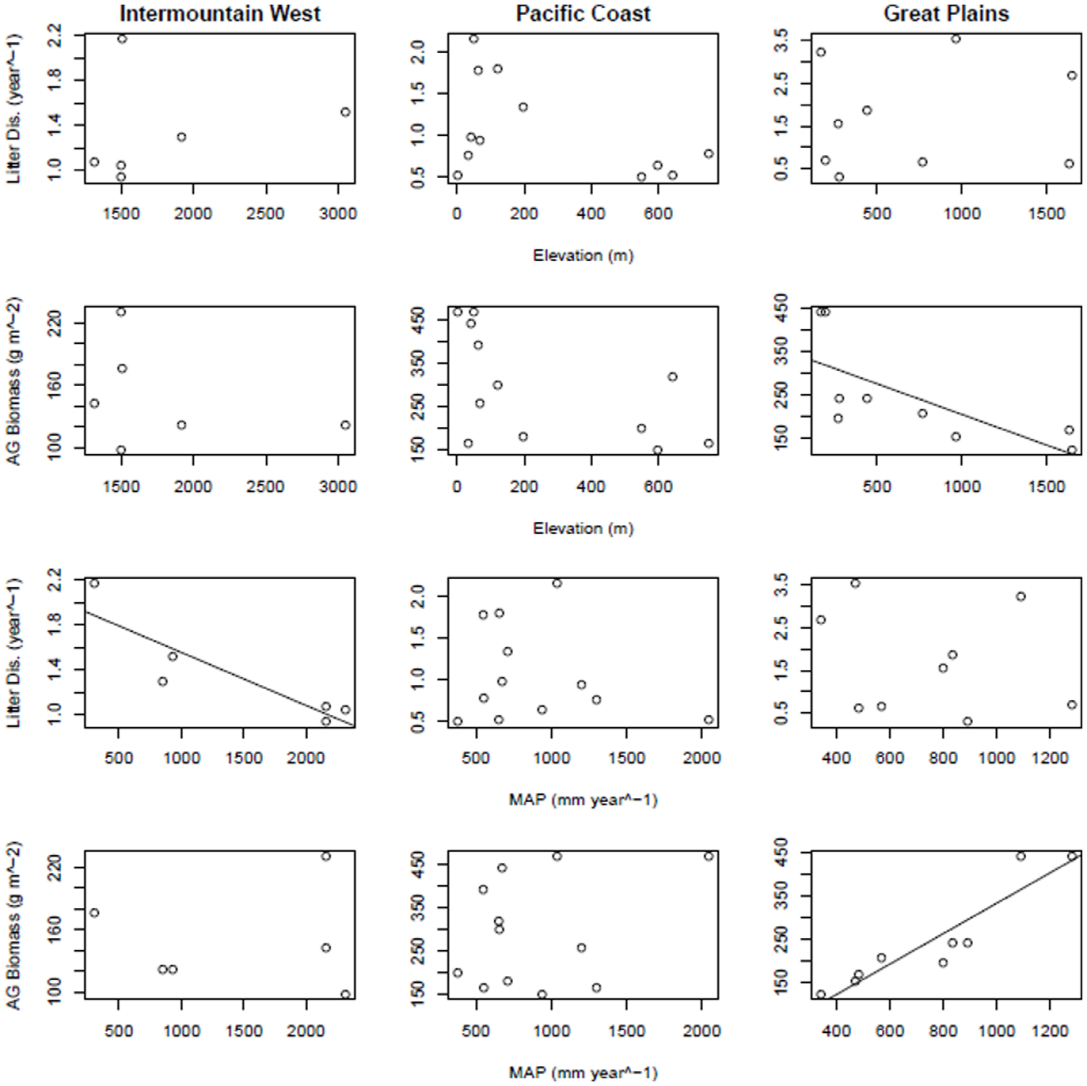
Site scale correlations between litter disappearance (Litter Dis.), aboveground biomass (AG Biomass), and physical variables (elevation and mean annual precipitation (MAP)) within three U.S. regions, Intermountain West, Pacific Coast, and Great Plains. Significant relationships are depicted by correlation lines; Intermountain West litter disappearance and precipitation (*p* = 0.02, *r^2^* = 0.74), Great Plains aboveground biomass and elevation (*p* = 0.03, *r^2^* = 0.44) and Great Plains aboveground biomass and mean annual precipitation (*p*<0.001, *r^2^* = 0.84).

Previous studies have found strong relationships among productivity, decomposition, and biophysical factors (*e.g.,* precipitation, soil chemistry) within regions (*e.g.,* U.S. Great Plains [40], [41]), so we examined relationships among productivity, litter and climate factors within three regions with sufficient replication in the U.S., Pacific Coast (n = 12), Intermountain West (n = 6), and Great Plains (n = 9). We found a significant negative correlation between litter disappearance and mean annual precipitation (*r^2^* = 0.71, *p* = 0.01) for the Intermountain West region (Figure 4). Sites in the Great Plains showed a positive relationship between aboveground biomass and precipitation (*r^2^* = 0.85, *p*<0.001) and a negative relationship between aboveground biomass and elevation (*r^2^* = 0.40, *p* = 0.02), although the strength of the latter relationship was much weaker (Figure 4).

## Discussion

In contrast to more commonly held perspectives that aboveground biomass production and decomposition processes should be positively correlated [3], we found inconsistent site-scale correlations between aboveground biomass and loss. Aboveground biomass, litter stocks, and litter disappearance varied depending on spatial scales, with aboveground biomass varying similarly at plot, site and continent scales, litter varying strongly among sites and litter disappearance varying strongly among continents. These results do not call into question the fundamental importance of temperature and precipitation for primary production or microbial decomposition, but rather indicate that their relative influences may vary, possibly due to differences in seasonality (*e.g.,* temperate vs. Mediterranean), interannual variability, and the strength of feedbacks between climate and factors including vegetation quality (*e.g.,* [4]. [6]), herbivory (*e.g.,* [14]), UV degradation (*e.g.,* [40], [21]), or nutrient cycling (*e.g.,* [42], [18], [20]).

Regional-scale analyses of grassland processes have found strong relationships between productivity, decomposition, and climatic variables (*e.g.,* [40], [41], [43]), but we found the relative intensity of these relationships can vary across grassland biomes. These previous studies were concentrated in the Great Plains region of the United States, and have served as the basis for assumptions of the generality of regional-scale coupling among these factors (*e.g.,* [44], [3]). Our data from this same region confirm a strong, positive relationship between aboveground biomass and mean annual precipitation. In other regions of the planet, however, there were substantial deviations. Similar regional-scale discrepancies have been reported previously in research on climate influences on net primary production. Knapp and Smith [34] reported no generalizable trend between variability in rainfall and production in 11 LTER sites in North America, but a broad-scale analysis of the same relationship in China found these factors to be tightly linked [45]. Our results demonstrate that aboveground biomass and litter disappearance do not necessarily covary nor are they always similarly controlled by climatic influences. Our results underscore the need for replication among regions and continents when characterizing live biomass-litter relationships, including their implication for global-scale carbon flux models.

While aboveground biomass and litter disappearance both varied at the site scale, the spatial scale of their variation was uncoupled at larger (*e.g.,* continent) and smaller (*e.g.,* plot) spatial scales. Further, while litter disappearance varied among sites and continents, it was not well-predicted by climate variables, suggesting that across widely distributed sites, neither process can be accurately predicted by regional climate. This is in contrast to the relationships found in previous studies between biomass production or decomposition rates (*k* values) and geographic and climatic factors, a discrepancy explained by the wider scope of our study and our simultaneous measurement of both factors (*e.g.*, [40], [46], [47], [7], [49], [48], [41]). One implication is that, at a global scale, temperature alone may not always accelerate the release of litter carbon to the atmosphere via decomposition, which has been a predicted effect of global warming [50]. Again, this does not contradict the fundamental importance of temperature in influencing decomposition, but suggests the impact of global temperature increases may vary regionally depending on the relative importance of other factors.

Radiation and latitude appear to influence the amount of biomass production at the site scale but were not related to the amount of litter or decomposition. This decoupling between production and decomposition processes is reinforced by the difference in spatial scales at which each process varies, pointing to likely drivers. The large-scale variation of decomposition is concordant with previous work showing decomposition as a function of temperature (although effects of temperature on organic matter can vary depending on quality, microbial community and enzymatic influences [51], soil moisture [52], leaf litter chemistry [53], [28], [29], [5], [6], actual evapotranspiration [1], leaf litter lignin [31] and microbial activity [54], all of which vary strongly among regions and continents. Because we focus on aboveground litter disappearance as a measure of decomposition, the relevance of these findings to belowground processes remains to be tested. In general, consistency in rates of decomposition between roots and shoots tends to depend on relative levels of recalcitrant carbon compounds and/or nutrients in the two tissue types; in some cases they are concordant [55], [5], whereas in other cases, roots tend to be more decay-resistant [4], [56]. For aboveground biomass, variability was evident among plots, sites and continents. This suggests that, in some regions, local factors such as small-scale variation in water or nutrient variability, species composition, herbivory or diversity [57]–[59] may constrain biomass production more than climatic factors.

There is increasing need for effective predictions of carbon cycle responses in grasslands, as mediated by production and decomposition, because of the importance of this biome to carbon pools, species diversity, and human livelihood. This is challenging because of the regional variation in projected shifts in temperature and precipitation associated with climate change [60]. Although carbon cycling models (*e.g.,* CENTURY model [8], [9]) assume that net primary production and decomposition are coupled via parallel responses to climatic factors, our results demonstrate that the relationship of these processes with climate can differ by region, and the dominant spatial scales of variation differ for grassland production and decomposition. While the CENTURY model was developed for the US Great Plains [8], [9], our empirical results suggest that effective long-term predictions of carbon flux will require a careful consideration of production and decomposition and should be applied with caution to other areas of the globe. In particular, carbon flux models that are regionally parameterized with flexible terms describing the independent strength and direction of production and decomposition with temperature and precipitation are likely to improve predictions of carbon dynamics in this globally important ecosystem.

Our study provides a succinct comparison of important herbaceous ecosystem functions: biomass production and litter loss across many geographical regions. Provided sufficient funding and spatial replication between sites, future studies over multiple growing seasons will contribute to this growing understanding of global divers in these systems. Future data from multiple years will allow us to capture interannual variability, an important component of herbaceous system carbon dynamics, not reflected in this dataset.Furthermore, a more comprehensive examination of nutrient and light availability and use in the context of biomass and litter measurements across grasslands worldwide will further explain global patterns in grassland carbon dynamics.
